# Somatic Loss of the Y Chromosome and Alzheimer’s Disease Risk

**DOI:** 10.1002/alz.090093

**Published:** 2025-01-03

**Authors:** Holly N. Cukier, Ellen L. Palmer, Penelope Benchek, Nicholas R. Wheeler, Sandra Smeisek, Adam C. Naj, Jonathan L. Haines, Margaret A Pericak‐Vance, Lars Forsberg, Yeunjoo E. Song, William S. Bush

**Affiliations:** ^1^ John P. Hussman Institute for Human Genomics, University of Miami Miller School of Medicine, Miami, FL USA; ^2^ Dr. John T. Macdonald Foundation Department of Human Genetics, University of Miami Miller School of Medicine, Miami, FL USA; ^3^ Department of Neurology, University of Miami Miller School of Medicine, Miami, FL USA; ^4^ Department of Population and Quantitative Health Sciences, Case Western Reserve University, Cleveland, OH USA; ^5^ Penn Neurodegeneration Genomics Center, Department of Pathology and Laboratory Medicine, Perelman School of Medicine, University of Pennsylvania, Philadelphia, PA USA; ^6^ Department of Population and Quantitative Health Sciences, Institute for Computational Biology, Case Western Reserve University, Cleveland, OH USA; ^7^ University of Miami Miller School of Medicine, Miami, FL USA; ^8^ Uppsala University, Uppsala Sweden

## Abstract

**Background:**

Mosaic loss of the Y chromosome (LOY) is a somatic, age‐related event that has been previously associated with a variety of diseases of aging. A prior study of European cohorts demonstrated an association between LOY and Alzheimer’s Disease and more recent molecular studies have shown that LOY can also occur within microglia, suggesting a potential functional role in AD pathogenesis.

**Method:**

In this study, we further validate the association between LOY in blood and AD via prospective analyses of 1,447 males. We defined a continuous estimate of LOY mosaicism by computing the median of the log R ratio (mLRRY) from raw intensity genotyping data. We also perform Mendelian Randomization analysis on 10,013 males across 26 US cohorts from the Alzheimer’s Disease Genetics Consortium (ADGC) dataset.

**Result:**

Age at blood draw was stratified into four bins consistent with prior literature (≤65, 66‐75, 76‐85, ≥86), and Wilcoxan rank sum tests were performed to examine the distribution of LOY by age at blood sampling. This trend of LOY increasing with age was highly significant over all samples and between all age groups. Within males with an available clinical diagnosis (n = 3117), there are significant differences between males ≤65 and all other age groups (66‐75 p = 0.00061, 76‐85 p = 7.9e‐12, ≥86 p = 5.5e‐13).

To evaluate the association between LOY and risk of developing AD in follow‐up, we fit a Cox proportional hazards model associating binary LOY to years of disease‐free follow‐up within 1,447 individuals for whom blood was sampled prior to any AD diagnosis. Of these, there were 42 subsequent AD events with all others remaining controls. Overall, we find a significantly increased hazard (1.98 [1.17–6.91], p = 0.0021) in men with LOY versus those without. To account for potential biases in sample ascertainment with respect to age and case/control status, we performed Mendelian Randomization analyses of LOY. Using the Wright‐based instrument, we had a significant two‐sample Mendelian randomization effect using the Inverse Variance Weighted approach (p = 0.008).

**Conclusion:**

Significant results from these analyses provide further evidence for a role of LOY in the development of Alzheimer’s Disease.

